# A Semi-Micro Extraction Spectrophotometric Determination of Iron Using 4-Nitrocatechol and Xylometazoline Hydrochloride

**DOI:** 10.3390/molecules30040899

**Published:** 2025-02-15

**Authors:** Petya V. Racheva, Antoaneta D. Saravanska, Galya K. Toncheva, Denitsa D. Kiradzhiyska, Nikolina P. Milcheva, Vidka V. Divarova, Ina P. Pencheva, Kirila T. Stojnova, Vassil B. Delchev, Kiril B. Gavazov

**Affiliations:** 1 Department of Chemical Sciences, Faculty of Pharmacy, Medical University of Plovdiv, 120 Buxton Bros Str., 4004 Plovdiv, Bulgaria; petya.racheva@mu-plovdiv.bg (P.V.R.); antoaneta.saravanska@mu-plovdiv.bg (A.D.S.); denitsa.kiradzhiyska@mu-plovdiv.bg (D.D.K.); nikolina.milcheva@mu-plovdiv.bg (N.P.M.); vidka.divarova@mu-plovdiv.bg (V.V.D.); 21301028@mu-plovdiv.bg (I.P.P.); 2 Department of General and Inorganic Chemistry with Methodology of Chemistry Education, Faculty of Chemistry, University of Plovdiv ‘Paisii Hilendarski’, 24 Tsar Assen St., 4000 Plovdiv, Bulgariastoynova@uni-plovdiv.bg (K.T.S.); 3 Department of Physical Chemistry, Faculty of Chemistry, University of Plovdiv ‘Paisii Hilendarski’, 24 Tsar Assen St., 4000 Plovdiv, Bulgaria; vdelchev@uni-plovdiv.net

**Keywords:** iron(III), spectrophotometric determination, 1,2-dihydroxy-4-nitrobenzene, xylometazoline hydrochloride, liquid-liquid extraction, B3LYP calculations

## Abstract

A chromogenic solvent extraction system for Fe(III) based on 4-nitrocatechol (4NC) and xylometazoline hydrochloride (XMH) was investigated. The optimum conditions for extraction spectrophotometric determination of iron were found. Under these conditions, the formula of the extracted complex was (XMH^+^)_3_[Fe(4NC)_3_] and the apparent molar absorption coefficient at *λ*_max_ = 464 nm was 1.9 × 10^5^ dm^3^ mol^−1^ cm^−1^ (4-fold enrichment). To validate the aforementioned formula and gather information about the spin state of the central Fe(III) ion, a combined experimental-theoretical approach was employed. This approach entailed the experimental determination of the 4NC:Fe and XMH:Fe molar ratios and the optimization of potential color-bearing structures utilizing the B3LYP/6-311G computational chemistry method. The effect of foreign ions was thoroughly examined, and a sensitive, simple, and inexpensive analytical procedure was proposed, which was successfully applied for determining iron in pharmaceutical and industrial samples.

## 1. Introduction

Iron is among the most extensively studied elements, with a long history in metallurgy, construction, catalysis, warfare, and numerous applications in everyday life. It is a critical component in a number of essential biological processes, including oxygen transport, electron transfer, and DNA synthesis or repair [1]. Although its homeostasis is tightly regulated, an imbalance—either a deficiency or an overload—can occur [2,3,4,5].

Iron can exist in several oxidation states, and a substantial body of research has been conducted on the complexation capacity of iron(II, III) toward catechol-type ligands. The impact of various substituents, including nitro groups, has been investigated with the objective of developing novel effective iron chelators [6,7], antioxidants [8], adsorption surfaces [9], COMT inhibitors [10], reactive species [11], superparamagnetic nanoparticles [12], atmospheric particulate matter [13,14], and complexation agents [15,16,17].

**Figure 1:**
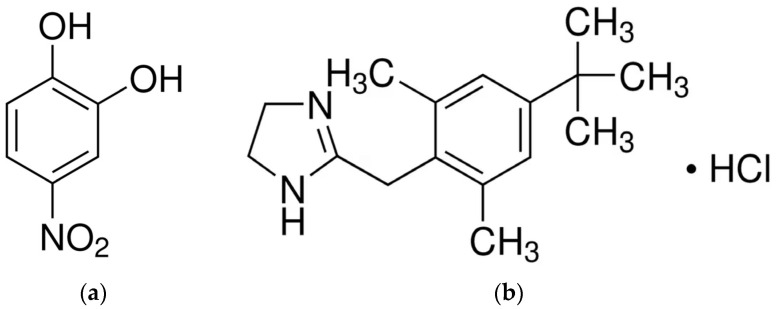
Structural formulas of 4NC (**a**) and XMH (**b**).

4-Nitrocatechol (4NC, H_2_L) (Figure 1a) occupies a significant position among the catecholic ligands. It is categorized as a non-hazardous substance according to the European Union’s Regulation (EC) No. 1272/2008, which classifies substances based on their potential to cause acute or chronic risks to humans and the environment [18]. The presence of a nitro substituent in the aromatic ring of 4NC enhances the acidity of the catechol function, thereby augmenting the complexing power with respect to other ortho-diphenols. This group may increase the ligand field, leading to greater splitting of d-orbitals and its propensity to form low-spin complexes [17]. The employment of 4NC in a variety of applications, including analytical chemistry [19,20,21,22], catalysis, and environmental remediation, underscores its versatility and significance. Notably, it serves as a biomarker for breast cancer, a model compound for studying how natural organic matter mobilizes and immobilizes metal ions, and is an important intermediate in the breakdown of nitrogen-containing compounds [22,23,24]. Despite its classification as one of the most significant organic analytical reagents [19], the application of 4NC in the determination of iron remains unexplored.

Nurchi et al. [6] have emphasized the intricate and dynamic nature of the UV-Vis spectra of Fe(III)–4NC species. However, the observed spectral changes have been attributed exclusively to a stepwise complexation process involving the formation of species with formulas FeL^+^, FeL_2_^−^, and FeL_3_^3−^, where L represents a doubly deprotonated 4NC anion (L^2−^ = H_2_L − 2H^+^) [6,15,25]. Notably, the extant literature contains no reports of protonated Fe(III)-4NC complexes containing HL^–^, a phenomenon observed in other ortho-diphenolic ligands [26]. Additionally, the spin state (low spin, high spin, or spin crossover) of the central Fe(III) ion remains undefined, a salient aspect in the context of the findings presented in [8,17,27,28].

The objective of the present study was to examine a liquid-liquid extraction (LLE) system comprising Fe(III), 4NC, xylometazoline hydrochloride (XMH), water, and chloroform. XMH (Figure 1b) has been utilized in a variety of fields and has been incorporated into the WHO Model List of Essential Medicines [29]. Within the domain of analytical chemistry, it has been employed as a colorless cationic ion-association reagent, capable of forming hydrophobic ternary complexes with intensely colored complex anions, as evidenced by several studies [24,30,31]. The present paper encompasses both experimental LLE–spectrophotometric studies and theoretical time-dependent density functional theory (TD DFT) calculations, alongside the simulation of theoretical UV-vis spectra of optimized putative structures and a comparison with experimental spectra. This approach has previously been employed in studies of 4NC complexes of Al(III) [23], V(IV,V) [24], Mo(VI) [20], and W(VI) [21] and has proven fruitful.

## 2. Results and Discussion

### 2.1. Extraction-Spectrophotometric Optimization

**Figure 2:**
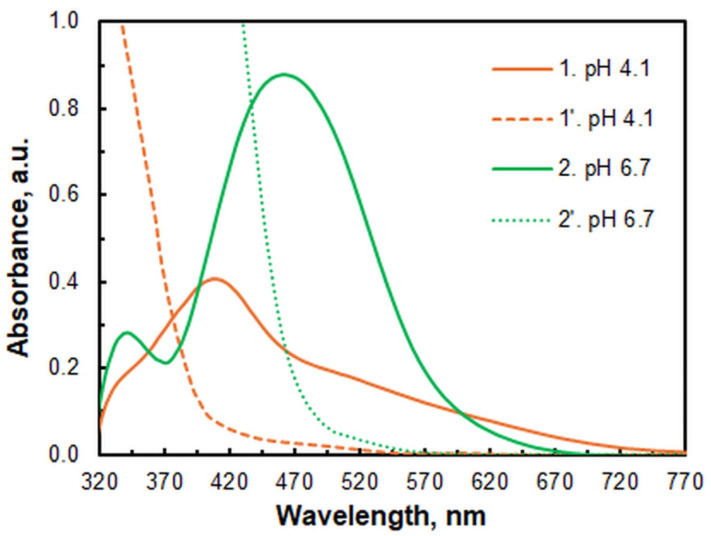
Absorption spectra in chloroform of the ternary species (1, 2) and corresponding blanks (1′, 2′) at different pH values: *c*_Fe_ = 4.6 × 10^−6^ mol dm^−3^, *c*_4NC_ = 2 × 10^−4^ mol dm^−3^, *c*_XMH_ = 7.5 × 10^−4^ mol dm^−3^, *t*_ex_ = 1 min.

Figure 2 compares the spectra of the extracted ternary species and the corresponding blanks at two different pH values. It can be concluded that at least two different complexes are formed. One is characterized by the maximum absorption wavelength (*λ*_max_) = 408 nm. The other one is formed at higher pH values and shows an absorption maximum at 464 nm.

**Figure 3:**
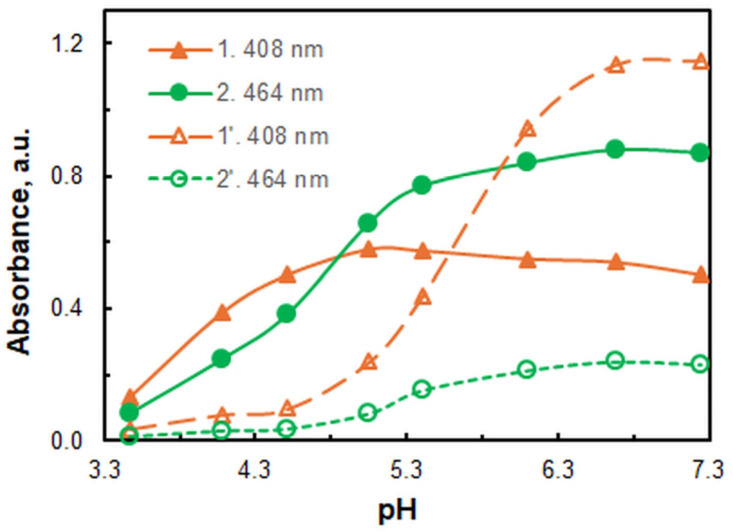
pH curves of the ternary species (1, 2) and corresponding blanks (1′, 2′) at two different wavelengths. *c*_Fe_ = 4.6 × 10^−6^ mol dm^−3^, *c*_4NC_ = 2 × 10^−4^ mol dm^−3^, *c*_XMH_ = 7.5 × 10^−4^ mol dm^−3^, and *t*_ex_ = 1 min. The pH was maintained using 3 cm^3^ of ammonium acetate buffer.

The effect of pH on the absorbance at these two wavelengths is shown in Figure 3. The intersection of the pH curves occurs at a pH close to 4.8, and the maximum absorbance is obtained at a pH of 6.7. A series of buffers prepared from 2 mol dm^−3^ solutions of acetic acid and ammonia were used to maintain pH. The effect of buffer volume (between 0.5 and 3.5 cm^3^) was also studied. The results demonstrated that the absorbance remained constant, indicating that the components of the buffer are not involved in complexation. Given the relatively limited buffering capacity at pH_opt_ = 6.7, the volume of 3 cm^3^ was selected for analytical purposes.

The minimum shaking time required to achieve maximum absorbance was approximately 30–40 s. The resulting absorbance remained constant when the shaking time (*t*_ex_) was increased to at least 5 min. The effect of longer extraction times on the process has not been investigated. To ensure stability of results and maintain operational efficiency, all subsequent experiments were conducted at a *t*_ex_ of 1 min.

**Figure 4:**
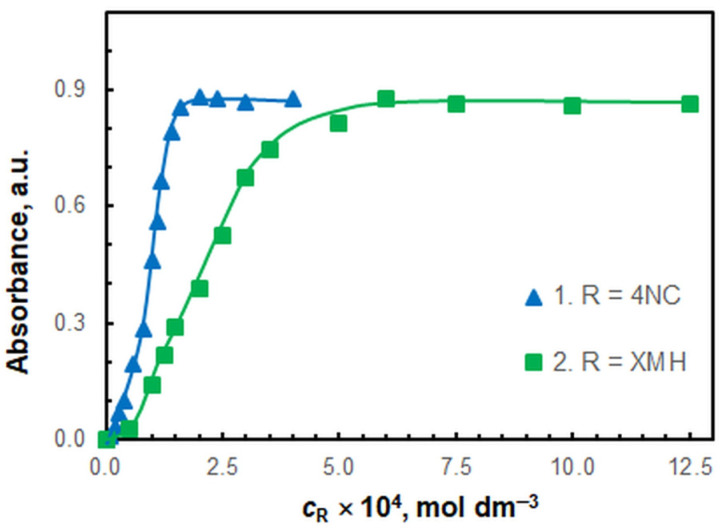
Effect of 4NC (curve 1) and XMH (curve 2) concentrations. *c*_Fe_ = 4.6 × 10^−6^ mol dm^−3^, pH = 6.7, *λ* = 464 nm, *t*_ex_ = 1 min. 1. *c*_XMH_ = 7.5 × 10^−4^ mol dm^−3^; and 2. *c*_4NC_ = 2.0 × 10^−4^ mol dm^−3^.

The effect of the concentrations of 4NC and XMH in the aqueous phase on the absorbance is shown in Figure 4. The following concentrations were chosen for further investigation: *c*_4NC_ = 2 × 10^−4^ mol dm^−3^ and *c*_XMH_ = 7.5 × 10^−4^ mol dm^−3^. The experimental series were conducted with chloroform volumes of 10 cm^3^ and 2.5 cm^3^. In alignment with environmental concerns, the well-being of laboratory personnel, sustainability, and the degree of greenness [32], the volume for the chloroform phase was set to 2.5 cm^3^.

Table 1 summarizes all optimized parameters. The optimization intervals and the optimal values found are included.

### 2.2. Molar Ratios and Suggested Formulas of the Extracted Species

Nurchi et al. [6] and Hakkinen [25] reported that Fe(III) and 4NC form three complexes in aqueous media, which can be represented by the following formulas: [FeL]^+^, [FeL_2_]^−^, and [FeL_3_]^3−^. The 1:3 complex is formed in excess of 4NC at pH levels above 5. This complex is distinguished by a composite band centered at 454 nm in an aqueous medium [6].

**Figure 5:**
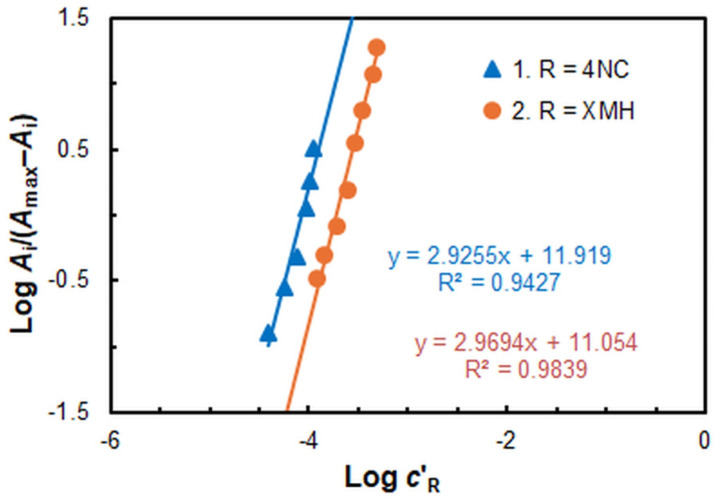
Determination of the 4NC:Fe (1) and XMH:Fe (2) molar ratios by the mobile equilibrium method.

The findings of extraction studies in the presence of XMH, processed by the mobile equilibrium method [33] (Figure 5, straight line 1) and the straight-line method of Asmus [34], corroborate the conclusion that the Fe:4NC molar ratio is 1:3. The aforementioned methods yield results indicating that the Fe:XMH molar ratio is also 1:3 when the pH was 6.7 (see Figure 5, straight line 2). Based on these findings, it can be postulated that the formula of the extracted ternary complex at pH_opt_ 6.7 is (XMH^+^)_3_[Fe^III^L_3_]. The discrepancy in the absorption maxima of the ternary complex in chloroform (464 nm) and the anionic chelate in an aqueous medium (454 nm) is negligible. It can be attributed to the influence of both the solvent and the ion-association reagent (XMH).

**Figure 6:**
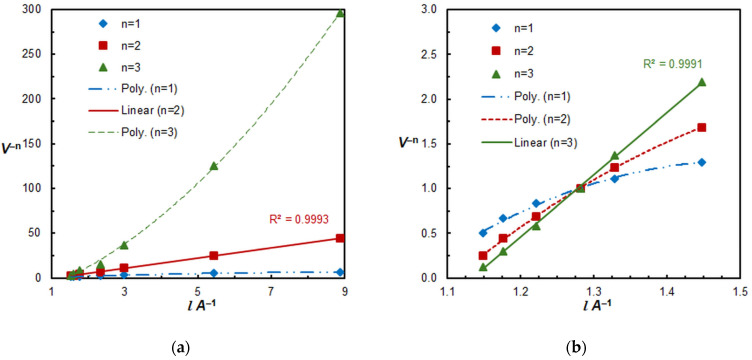
Determination of the XMH:Fe molar ratios by the straight-line method of Asmus. *c*_4NC_ = 4 × 10^−4^ mol dm^−3^, pH 5.3 (**a**) at low XMH concentrations (up to 3.5 × 10^−4^ mol dm^−3^), and (**b**) at higher XMH concentrations.

The results obtained at a pH of 5.3 revealed the presence of two distinct ternary complexes, contingent on the concentration of XMH (see Figure 6). At a low XMH concentration, a 1:2 Fe:XMH complex is formed (see Figure 6a), which most likely contains a singly protonated 4NC ligand and can be represented by the formula (XMH^+^)_2_[Fe(HL)L_2_].

**Figure 7:**
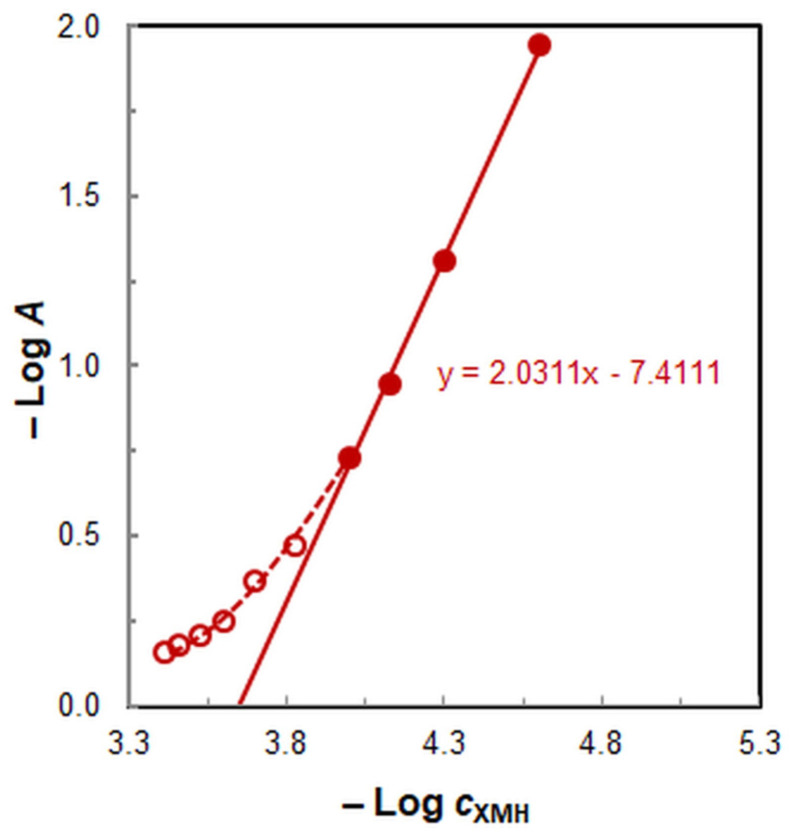
Determination of XMH:Fe molar ratio by the Bent–French limited logarithm method.

The Bent–French limited logarithm method [35] (Figure 7) lends further credence to the existence of this complex, as it yields reliable results only at low ligand concentrations.

### 2.3. Ground-State Equilibrium Geometries of Putative Anionic Structures

The spectral bands observed in the visible region of typical ion-association complexes containing colorless cations (e.g., XMH^+^) are attributed to the anionic part of the complex. This peculiarity has been utilized to elucidate the stoichiometry and geometry of 4NC complexes of vanadium [24], molybdenum [20], and tungsten [21] by comparing theoretical spectra of the anionic moiety with experimental spectra of the extracted ternary species.

**Figure 8:**
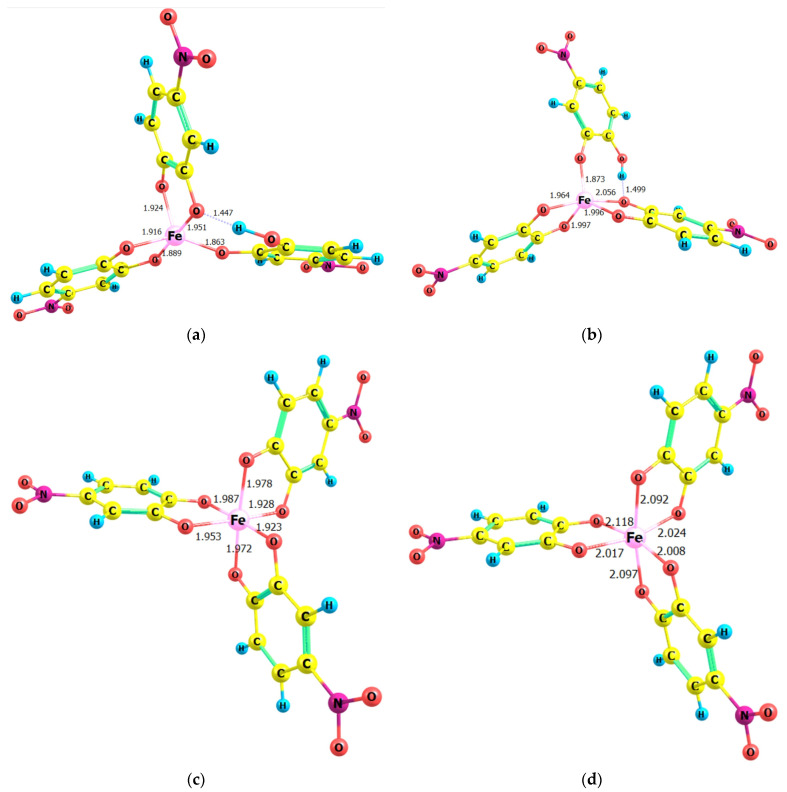
Optimized ground-state equilibrium geometries of some putative structures found at the B3LYP/6-311G level of theory. (**a**) Structure 1: [Fe(HL)L_2_]^2−^; charge = −2, multiplicity = 2. (**b**) Structure 2: [Fe(HL)L_2_]^2−^; charge = −2, multiplicity = 6. (**c**) Structure 3: [FeL_3_]^3−^; charge = −3, multiplicity = 2. (**d**) Structure 4: [FeL_3_]^3−^; charge = −3, multiplicity = 6.

In this regard, models of the anionic constituents of some putative structures that are consistent with the experimentally determined mole ratios were constructed and optimized at the theoretical B3LYP/6-311G level (Gaussian 03 software). The calculations were conducted under the assumption that the complex anions, [Fe(HL)L_2_]^2–^ and [FeL_3_]^3–^, would adopt either a low-spin (multiplicity = 2) or a high-spin (multiplicity = 6) configuration. The optimization results are presented in Figure 8.

As can be observed, the bond lengths in the coordination polyhedra of the low-spin structures (**a** and **c**) are shorter, and the polyhedra themselves (tetragonal pyramid for the protonated complexes and octahedron for the deprotonated complexes) are more regular. For instance, the O–Fe–O angle involving the axial oxygen atoms in the octahedral structures is more closely aligned with 180° in the low-spin configuration (175.50° vs. 170.17°). Furthermore, the displacement of the fourth oxygen atom from the plane of the square base of the pyramid in the protonated anion [Fe(HL)L_2_]^2−^ is less for the low-spin configuration (dihedral angles close to 20° vs. dihedral angles close to 29°).

It is noteworthy that the benzene nuclei of 4NC maintain their aromatic character in the complexes. This can be determined by the observation that the distances between carbon atoms in these nuclei remain comparable to those reported by Cornard et al. [16] for unbound H_2_L and to those in the stable complexes of 4NC with Mo [20] and V [24]. Consequently, the formation of typical quinoidal structures is not observed.

### 2.4. Simulated Spectra and Spectral Comparison

**Figure 9:**
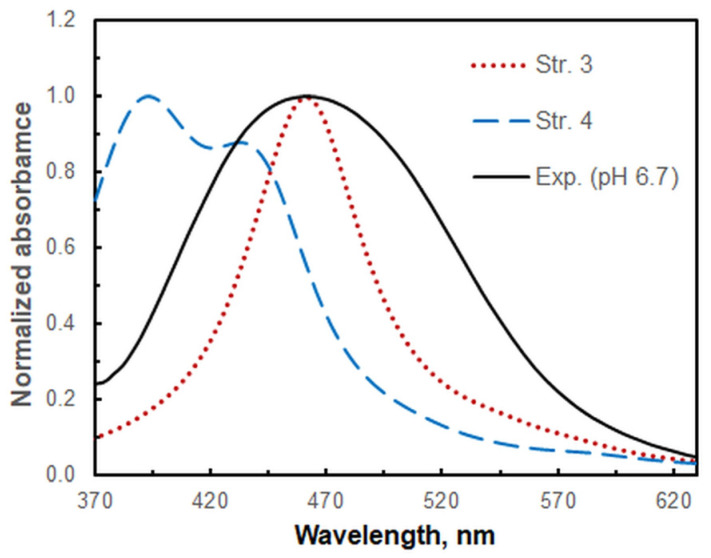
Comparison of the experimental spectrum at the optimum conditions (Exp.) and the theoretical spectra of the low-spin structure 3 and the high-spin structure 4. A scaling coefficient of 0.79 and a Lorentzian broadening were used for the theoretical spectra.

The optimized structures depicted in Figure 8 were employed to calculate vertical excitation energies with the time-dependent Hamiltonian, with the objective of simulating theoretical absorption spectra. The calculated spectral line locations of the four modeled structures are presented in Table 2. As demonstrated in Figure 9, the application of a scaling coefficient of 0.79 leads to a satisfactory alignment between the experimental spectrum under optimal extraction conditions (Exp.) and the theoretical spectrum of the low-spin structure 3 (Str. 3). Conversely, the spectrum of the high-spin structure 4 (Str. 4) does not align with the experimental spectrum under any scaling conditions. Consequently, it can be deduced that the low-spin electron configuration (single d electron, multiplicity = 2) is realized in the complexation under the optimum conditions.

### 2.5. Extraction Characteristics

The findings from both experimental and theoretical studies suggest that under optimal conditions, the complex formation and the subsequent extraction of the ternary complex can be described by the following equations:

Fe^III^_(aq)_ + 3 H_2_L_(aq)_ ≡ [Fe^III^(L)_3_]^3−^_(aq)_ + 6 H^+^_(aq)_ (1)

[Fe(L)_3_]^3−^_(aq)_ + 3 XMH^+^_(aq)_ ≡ (XMH^+^)_3_[Fe(L)_3_]_(chloroform)_ (2)

The extraction constant was calculated by two different methods based on an XMH saturation curve at the optimal 4NC concentration and pH. The methods employed were (1) the Holme–Langmyhr method [36] and (2) the mobile equilibrium method [33]. As shown in Table 3, the values obtained are statistically indistinguishable. Additionally, Table 3 presents the values of the distribution coefficient (*D*) and the fraction extracted (%*E*) determined spectrophotometrically by a known procedure [37].

### 2.6. Calibration Graph and Analytical Characteristics

The relationship between the measured absorbance and iron(III) mass concentration was found to be linear up to 0.27 µg cm^−3^ Fe(III) (*R*^2^ = 0.998). The study design involved a 4-fold enrichment, with the volume of the aqueous phase being 10 cm^3^ and that of the organic phase being 2.5 cm^3^. The linear regression equation was *A* = 3.40*γ* + 0.005, where γ is the concentration in µg cm^−3^. The standard deviation of the slope was determined to be 0.005, while that of the intercept was 0.006, indicating that the intercept is statistically indistinguishable from zero. The apparent molar absorptivity coefficient was found to be 1.90 × 10^5^ dm^3^ mol^−1^ cm^−1^, and the Sandall’s sensitivity was 2.94 × 10^−4^ µg cm^−2^. The limit of detection (LOD) and the limit of quantitation (LOQ) were calculated as 3 and 10 times the standard deviation of the blank (*n* = 10) divided by the slope, respectively, yielding values of 5.3 ng cm^−3^ and 18 ng cm^−3^.

The calculated molar absorptivity coefficient at equal volumes of the two phases (10 cm^3^) was approximately four times smaller: 4.8 × 10^4^ dm^3^ mol^−1^ cm^−1^.

### 2.7. The Effect of Foreign Ions

As demonstrated in Table 4, the presence of substantial quantities of common anions, such as Cl^−^, SO_4_^2−^, NO_3_^−^, and F^−^, is permissible without imposing constraints on the method of sample dissolution. Alkali and alkaline earth cations, as well as Zn(II) and Mn(II), also do not induce adverse effects when present in high concentrations. The most substantial interferences are caused by Al(III), which enhances the absorption significantly. Other ions capable of forming stable complexes with 4NC and thereby interfering with the analysis include Mo(VI), Ni(II), V(V), and W(VI).

### 2.8. Analytical Application

The procedure was applied to determine the iron content in Maltofer^®^ Fol tablets, which are marketed as containing 100 mg of iron(III) per tablet. The result, 103 ± 4 (mean ± standard deviation, four replicate analyses), was statistically identical to this value. To assess the inter-day reproducibility, eight replicate analyses were executed on the subsequent two days (four analyses per each day). The results from the three-day period demonstrated adequate reproducibility, with an average value of 102 mg and a relative standard deviation (RSD) of 4.6%. Subsequently, the range of analyses was expanded through the processing of industrial samples (Wälz oxides and calcium carbonate) supplied by a metallurgical plant engaged in the production of zinc and other metals. The results are presented in Table 5.

### 2.9. Comparison with Existing Methods

A comparative analysis of the present method with other solvent extraction spectrophotometric methods for the determination of Fe(II,III) ions is provided in Table 6. The present method demonstrates sensitivity, as evidenced by the calculated molar absorptivity (1.9 × 10^5^ dm^3^ mol^−1^ cm^−1^) and LOD (5.3 ng cm^−3^). The method is reliable and robust, as the optimal intervals for the examined parameters are sufficiently broad. The reagents employed are commercially available and cost-effective. Therefore, in contrast to the extant literature [38,39,40,41,42,43], the synthesis of reagents is not required. The equipment is simple, and all operations can be considered routine, thus eliminating the need for highly trained personnel.

The time required to complete the analysis is shorter than that reported for other methods [39,44,45,46,47,48]. The prolonged duration observed in these methods can be attributed to various factors, including extended equilibration times [44,47], a challenging phase separation process [44,49], the necessity of drying the organic layer [4,45,46], or the inclusion of labor-intensive steps following extraction, which are essential for the formation of the colored complex [39,50].

**Table 6:** A comparison of different methods for determining Fe ions using solvent extraction and spectrophotometry.

Reagent(s)	Technique	Organic Solvent	pH	Working Range, μg cm^−3^	*λ*, nm	10^−4^*ε*, dm^3^ mol^−1^ cm^−1^	Sample	Ref.
DBA	LLE	n-Butanol	8.2	–	430	0.5	Commercial mixtures, pharmaceutical samples and alloys	[51]
DHN	LLE	Ethyl acetate	5–8	0.05–10	485	1.1	Silicate rock samples	[52]
DHN + CTAB	LLE	Ethyl acetate	8–10	0.04–5.0	455	1.2	Geological samples, concentrates, tobacco leaves, cigarettes, and waters	[26]
DM + HAs	LLE	Chloroform	5.3–7.2	0.03–4.2	552–583	3.08–4.33	Pharmaceutical, biological, and food samples	[47]
DPCP	LLE	Chloroform	3.5–4	0.5–10	525	0.1156	Pharmaceutical samples	[38]
DPTP + KSCN	LLE	Chloroform, acetone	6.0	–	480	–	Steel sample	[39]
GA + CTAB	DLLME	C_2_Cl_4_ + acetone	5.0	0.05–0.65	560	_	Water and food samples	[53]
HHTPs + HAs	LLE	Chloroform	3.5–6.0	0.40–22.5	545–595	3.0–4.5	Oil and oil products	[48]
KSCN + CTAB (TPPC)	LLE	Chloroform	0.2 mol dm^−3^ H_2_SO_4_	0.056–2.24	473 (506)	3.55	–	[44]
KSCN + CTAB	LLE	Ethyl acetate	3–4	Up to 6.0	474	3.2	Geological and hydrogeochemical samples	[45]
KSCN + DMF	LLE	Benzene	5 mol dm^−3^ H_2_SO_4_	0.05–12	490	7.2	Model and industrial solutions	[54]
MPDDO	LLE	Dichloromethane	2.0	1–10	235	0.303	Synthetic mixtures	[46]
NH_4_SCN + N-OOA	LPME-NDS	Chloroform	Strong acidic solution	0.05–6.0	477	18.0	Food, biological, and environmental samples	[4]
NSTS	LLE	Isoamyl alcohol	4.0–6.0	1–7	435	2.88	–	[55]
PHEN + AA	EIEB	–	4.5	0.050–2.0	510	–	Diesel oil	[49]
TBP + DB18C6, NH_4_SCN	LLE	Kerosene	0.01 mol dm^−3^ CH_3_COONa	–	475	–	Soap samples	[50]
4NC + XMH	LLE	Chloroform	6.7	0.018–0.27	464	19.0	Pharmaceutical and industrial samples	This work

Abbreviations: 4NC, 4-nitrocatechol; AA, ascorbic acid; CTAB, cetyltrimethylammonium bromide; DB18C6, dibenzo-18-crown-6; DBA, 2, 4-dimethyl-3H-1,5-benzodiazepine; DLLME, dispersive liquid-liquid microextraction; DM, dimercaptophenole; DMF, dimethylformamide; DPCP, 1,3-diphenyl-4-carboethoxy pyrazole-5-one; DPTP, 1,5-dimethyl-2-phyenyl-4[(E)-(2,3,4-trihydroxy phenyl)diazenyl]-1,2-dihydro-3H-pyrazole-3-one; DHN, 2,3-dihydroxynaphthalene; EIEB, extraction induced by emulsion breaking; GA, gallic acid; HAs, hydrophobic amines; HHTPs, hydroxyhalogenthiophenols; LLE, liquid-liquid extraction; LPME-NDS, liquid-phase microextraction nanodrop spectrophotometry; MPDDO, 4-methyl-2,3-pentanedione dioxime; N-OOA, N-octylacetamide; NSTS, 5-nitrosalicylaldehyde thiosemicarbazone; PHEN, 1,10-phenanthroline; TBP, tributyl phosphate; TPPC, tetraphenylphosphonium chloride; XMH, xylometazoline hydrochloride.

## 3. Materials and Methods

### 3.1. Chemicals and Instruments

An iron(III) stock solution was prepared from ammonium iron(III) sulfate dodecahydrate (Merck, ACS reagent, 99%, Schnelldorf, Germany) in accordance with the procedures outlined in reference [26,56]. The working solutions were obtained by suitable dilution of the stock solution with water.

A 2 × 10^−3^ mol dm^−3^ aqueous solution of 4NC (>98%, Fluka AG, Buchs, Switzerland) was prepared by dissolving 0.9695 g of the reagent in a 250 cm^3^ calibrated flask.

A solution of XMH at a concentration of 5 × 10^−3^ mol dm^−3^ (250 cm^3^) was prepared by thoroughly dissolving 0.3511 g of the reagent (≥99%, Merck, Schnelldorf, Germany) in water. Following preparation, the solution was stored in a dark glass vessel [30].

The acidity of the aqueous medium was established through the use of a buffer solution, which was prepared by combining 2 mol dm^−3^ aqueous solutions of CH_3_COOH and NH_4_OH. The pH was subsequently verified using an InoLab 720 pH meter (WTW, Weilheim, Germany). Distilled water was used during the experimental process, and chloroform was used repeatedly after pre-distillation. A bottle-top dispenser (Ceramus Classic, Hirschmann, Germany) was used to introduce chloroform into the separating funnel.

Two UV/Vis scanning spectrophotometers were employed to measure the absorbances of the samples: an Ultrospec 3300 pro (Little Chalfont, UK) and a Drawell DU-8800RS (Chongqing, China). Both instruments were equipped with 10 mm macro- and semi-micro cuvettes.

### 3.2. Samples and Sample Preparation

Maltofer^®^ Fol (Vifor Pharma, France) chewable tablets, formulated for the treatment and prevention of iron deficiency, were procured from a local pharmacy. According to the product leaflet, each tablet contains 100 mg of iron(III). The tablets were prepared for analysis by following the procedure outlined in [57,58], which involves heating in a mixture of concentrated nitric acid (10 cm^3^) and concentrated sulfuric acid (1 cm^3^) on a sand bath.

Industrial samples (Wälz oxides and calcium carbonate) were supplied in powder form by KCM SA, a non-ferrous metallurgical plant located near Plovdiv, Bulgaria. Two samples of calcium carbonate (0.5 g) of different provenance were dissolved in 10 cm^3^ sulfuric acid (*w* = 10%) at gentle heating. The resulting solutions were filtered and transferred to 100 cm^3^ flasks. A few drops of 0.02 mol dm^–3^ KMnO_4_ were added before the solutions were made up to the mark with water, resulting in a stable violet color for a few minutes. Wälz oxides (0.1 g) were dissolved using hydrochloric acid (10 cm^3^, *w* = 15%) and hydrogen peroxide (1 cm^3^, *w* = 30%). The latter was added dropwise during the heating process. After filtration, the solution was collected in a 100 cm^3^ flask.

### 3.3. Optimization Procedure

Solutions of Fe(III), 4NC, XMH, and buffer were combined in a separatory funnel, and the total volume was adjusted to 10 cm^3^ with water. Then, chloroform (2.5 cm^3^) was added, and the mixture was shaken for extraction. A portion of the organic phase was poured into the cuvette, and the absorbance was measured against a similarly prepared blank.

### 3.4. Determination of the Extraction Characteristics

The constant of extraction (*K*_ex_) was calculated by two methods (the Holme–Langmyhr method and the mobile equilibrium method) based on different points from the saturation curve with XMH at the optimal pH and *c*_4NC_. The volume of the two phases was set equal to 10 cm^3^. All extraction experiments were conducted at room temperature.

The distribution ratio (*D*) was determined using the formula *D* = *A*_1_/(*A*_3_ − *A*_1_), where *A*_1_ is the measured absorbance after a single extraction and *A*_3_ is the absorbance of the pooled extracts obtained after triple extraction [21,37]. The fraction extracted (*E*) was then found by the equation %*E* = 100 × *D*/(*D* + 1).

### 3.5. Procedure for the Determination of Iron(III)

An aliquot of the analyzed solution containing 0.2–2.7 µg Fe(III) was placed in a separating funnel, to which 1 cm^3^ of a 2 ×10^−3^ mol dm^−3^ 4NC solution, 1 cm^3^ of a 7.5 × 10^−3^ mol dm^−3^ XMH solution, and 3 cm^3^ of a buffer solution (pH 6.7) were added. The volume of the aqueous phase was then adjusted to 10 cm^3^, 2.5 cm^3^ of chloroform was dispensed, and the mixture was shaken for one minute. Following phase separation, a portion of the chloroform layer was poured into a semi-micro cuvette (0.7 cm^3^ volume), and the absorbance was measured at 464 nm against a blank sample. The Fe(III) concentration was calculated using a calibration line.

### 3.6. Theoretical Calculations

The ground state geometries of the color-bearing anion components of the ternary complexes were optimized at the B3LYP level of theory, utilizing 6-311G basis functions. The spin multiplicities of the two- and three-charge complexes were set to 2 or 6, indicating that 1 or 5 single electrons of Fe(III) were distributed among the split 3d atomic orbitals. To ascertain the absence of imaginary frequencies in the optimized structures, frequency calculations were conducted, thereby confirming their stability as minima. Furthermore, vertical excitation energies were computed to simulate UV-Vis spectra. The calculations were executed using the Gaussian 03 software [59], and the ChemCraft graphical program [60], version 1.8, was employed for visualization of the optimized low- and high-spin structures.

## 4. Conclusions

An LLE system for Fe(III) was investigated through a combined experimental and theoretical approach, and the optimal conditions for a rapid, simple, sensitive, and robust semi-micro extraction-spectrophotometric determination of Fe(III) were found. The basis of the developed procedure is an intensely colored low-spin complex, which can be represented by the formula (XMH^+^)_3_[Fe^III^(4NC)_3_].

## Figures and Tables

**Table 1:** Extraction-spectrophotometric optimization *.

Parameter	Optimization Range	Optimal Value
Wavelength, nm	UV/Vis	464
pH	3.5–7.2	6.7
Volume of the buffer, cm^3^	0.5–3.5	3.0
Concentration of 4NC, mol dm^−3^	(0.1–6.0) × 10^−4^	2.0 × 10^−4^
Concentration of XMH, mol dm^−3^	(0.25–15) × 10^−4^	7.5 × 10^−4^
Shaking time, s	5–300	60
Volume of chloroform, cm^3^	2.5 and 10	2.5

* The experiments were conducted at room temperature (22 °C).

**Table 2:** Calculated wavelengths of the anionic structures (Str. 1–4). The *f* denotes the oscillator strength.

Str. 1 (Low-Spin)		Str. 2 (High-Spin)		Str. 3 (Low-Spin)		Str. 4 (High-Spin)	
*λ*, nm	*f*	*λ*, nm	*f*	*λ*, nm	*f*	*λ*, nm	*f*
1027	0.0015	740	0.0043	1325	0	738	0.0331
885	0.0003	690	0.0005	1190	0	695	0.0193
875	0.0005	659	0.0001	950	0	672	0.0126
861	0.0001	643	0.0063	771	0	653	0.0219
819	0.0035	615	0.0022	731	0.0076	639	0.0597
810	0.0001	591	0.0015	718	0	630	0.0049
679	0.0007	576	0.0011	695	0.0006	629	0.0007
657	0.0035	567	0.0013	690	0.0014	627	0.0027
644	0.0201	565	0.1092	690	0.0116	626	0.0283
606	0.0028	549	0.0758	672	0.0014	620	0.0232
603	0.0134	543	0.0213	660	0.0006	620	0.0207
596	0.0065	525	0.0008	655	0.0014	618	0.0144
575	0.0494	512	0.0145	655	0.0014	615	0.0023
571	0.0012	510	0.0059	649	0.001	598	0.0372
563	0.0282	508	0.0072	644	0.0015	595	0.0412
555	0.008	504	0.0691	636	0.0013	572	0.0081
548	0.0527	497	0.0192	624	0.0059	569	0.0333
524	0.0515	491	0.0351	590	0.1689	552	0.1013
517	0.0173	489	0.0615	581	0.1269	542	0.0453
514	0.0437	478	0.0473	578	0.0288	536	0.0071
509	0.0017	477	0.0072	564	0.038	522	0.0097
505	0.0184	471	0.0055	509	0.0005	514	0.0209
503	0.0019	464	0.0019	505	0.0028	507	0.0262
481	0.0036	450	0.012	495	0.0009	498	0.0006
		446	0.0004			491	0.0009
		445	0.0018			489	0.0015
		444	0.0036			485	0.0005

**Table 3:** Extraction characteristics.

Characteristic	Value
Extraction constant (log*K*_ex_)	11.14 ± 0.07 ^a^ (*n* = 4); 11.2 ± 0.4 ^b^ (*n* = 8)
Distribution ratio (log *D*)	1.38 ± 0.23 (*n* = 4)
Fraction extracted (%*E*)	95.7 ± 1.7 (*n* = 4)

^a^ Holme–Langmyhr method; ^b^ Mobile equilibrium method.

**Table 4:** Impact of foreign ions on the determination of 1.3 μg Fe(III) ^a^.

Foreign Ion (FI)	Formula of the Added Salt	Amount of FI Added/mg	FI:Fe(III) Mass Ratio	Amount of Fe Found/μg	*E*%
Al(III)	Al_2_(SO_4_)_3_·18H_2_O	1.3 × 10^−3^	1	2.39	184
Ba(II)	BaCl_2_	2.6	2000 ^b^	1.24	95.2
Ca(II)	CaSO_4_·2H_2_O	1.3	1000 ^b^	1.29	99.0
Cd(II)	CdSO_4_ ·8⁄3H_2_O	0.13	100	1.37	105
Cl^−^	NaCl	13	10,000 ^b^	1.28	98.2
Co(II)	CoSO_4_·7H_2_O	0.65	500	1.28	98.6
Cr(III)	Cr_2_(SO_4_)_3_	0.325	250	1.35	104
Cr(VI)	K_2_CrO_4_	1.3 × 10^−2^	10	1.33	102
Cu(II)	CuSO_4_·5H_2_O	6.5 × 10^−3^	5	1.29	99.5
F^−^	NaF	13	10,000 ^b^	1.31	101
Hg(I)	Hg_2_(NO_3_)_2_	0.325	250	1.25	96.4
Hg(II)	Hg(NO_3_)_2_·H_2_O	0.325	250	1.22	94.2
HPO_4_^2−^	Na_2_HPO_4_·12H_2_O	0.13	100	1.30	100
I^−^	KI	0.13	100	1.22	93.7
Mg(II)	MgSO_4_·7H_2_O	13	10,000 ^b^	1.28	98.6
Mn(II)	MnSO_4_·H_2_O	1.3	1000	1.29	99.4
Mo(VI)	(NH_4_)_6_Mo_7_O_24_·4H_2_O	1.3 × 10^−3^	1	1.42	109
Ni(II)	NiSO_4_·7H_2_O	1.3 × 10^−3^	1	1.40	108
NO_3_^−^	NaNO_3_	13	10,000 ^b^	1.24	95.3
Pb(II)	Pb(NO_3_)_2_	0.65	500	1.26	97.0
ReO_4_^−^	NH_4_ReO_4_	1.3	1000	1.33	102
SO_4_^2−^	K_2_SO_4_	13	10,000 ^b^	1.31	101
Tartrate	K,NaC_4_H_4_O_6_	1.3	1000	1.37	105
V(V)	NH_4_VO_3_	1.3 × 10^−3^	1	1.25	96.5
W(VI)	Na_2_WO_4_	1.3 × 10^−3^	1	1.38	106
Zn(II)	ZnSO_4_·7H_2_O	2.6	2000 ^b^	1.25	96.3

^a^ The volume ratio (*V*_aq_/*V*_chlorofom_) was 10/2.5 = 4. ^b^ Higher FI-to-Fe(III) mass ratios were not studied.

**Table 5:** Determination of iron in industrial samples.

#	Sample	Mass Fraction of Iron (*w*_Fe_) ^a^, %	Other Ingredients ^a^	*w*_Fe_ Determined ^b,c^, %	RSD, %
1	Wälz oxides	2.55	62.5% Zn, 9.25% Pb, 0.6% Cd, 0.25% Cl, 0.013% F, 0.04% As, 160 µg g^−1^ Sb, 16 µg g^−1^ Te, 10 µg g^−1^ Ge, 10 µg g^−1^ Se	2.51 ± 0.11	4.4
2	CaCO_3_	0.095	38.75% Ca, 0.13% Mg, 0.9% SiO_2_	0.098 ± 0.004	4.1
3	CaCO_3_	0.040	39.1% Ca, 0.12% Mg, 0.3% SiO_2_	0.039 ± 0.002	5.1

^a^ Determined in an alternate laboratory. ^b^ Mean ± standard deviation. ^c^ Four replicate determinations.

## Data Availability

Data are contained within the article.
